# Monitoring Master Swimmers’ Performance and Active Drag Evolution along a Training Mesocycle

**DOI:** 10.3390/ijerph18073569

**Published:** 2021-03-30

**Authors:** Henrique P. Neiva, Ricardo J. Fernandes, Ricardo Cardoso, Daniel A. Marinho, J. Arturo Abraldes

**Affiliations:** 1 Department of Sport Sciences, University of Beira Interior, 6201-001 Covilhã, Portugal; marinho.d@gmail.com; 2 Research Centre in Sports, Health and Human Development, CIDESD, 6201-001 Covilhã, Portugal; 3 Centre of Research, Education, Innovation and Intervention in Sport, Faculty of Sport, University of Porto, 4200-450 Porto, Portugal; ricfer@fade.up.pt (R.J.F.); ricardocardoso.coach@gmail.com (R.C.); 4 Porto Biomechanics Laboratory, University of Porto, 4200-450 Porto, Portugal; 5 Research Group MS&SPORT, Faculty of Sport Science, University of Murcia, 30720 San Javier, Spain; abraldes@um.es

**Keywords:** swimming, training control, biomechanics, speed, power

## Abstract

This study aimed to analyze the effects of a swimming training mesocycle in master swimmers’ performance and active drag. Twenty-two 39.87 ± 6.10 year-old master swimmers performed a 25 m front crawl at maximal intensity before and after a typical four-week training mesocycle. Maximum, mean and minimum speeds, speed decrease and hip horizontal intra-cyclic velocity variation were assessed using an electromechanical speedometer, and the active drag and power to overcome drag were determined using the measuring active drag system. Maximum, mean and minimum front crawl speeds improved from pre- to post-training (mean ± 95% CI: 3.1 ± 2.8%, *p* = 0.04; 2.9 ± 1.6%, *p* = 0.01; and 4.6 ± 3.1%, *p* = 0.01; respectively) and the speed decrease along the 25 m test lowered after the training period (82.5 ± 76.3%, *p* = 0.01). The training mesocycle caused a reduction in the active drag at speeds corresponding to 70% (5.0 ± 3.9%), 80% (5.6 ± 4.0%), and 90% (5.9 ± 4.0%), but not at 100% (5.9 ± 6.7%), of the swimmers’ maximal exertions in the 25 m test. These results showed that four weeks of predominantly aerobic training could improve master swimmers’ performance and reduce their hydrodynamic drag while swimming mainly at submaximal speeds.

## 1. Introduction

Competitive events for master swimmers have become increasingly popular over the years, aiming both for health [1] and performance benefits [2]. As a result, the number of master swimmers and their competitiveness level increased considerably in the last decade, with many participating in the last European and World Championships [3,4]. A better understanding of master swimming performance development along the training process is very important, but studies on the topic are rare [5,6,7]. Research has been mainly cross-sectional (making it difficult to extrapolate cause–effect relationships over time), and the traditionally conducted biomechanical analysis only focuses on the general kinematic variables (stroke frequency and length) and propelling efficiency [4,6,7,8]. Data often confirm that master swimmers display worse technical proficiency than elite swimmers [6,8].

Swimmers’ speed, which depends directly on the relationship between propulsive and hydrodynamic drag forces, defines the swimming performance [9,10,11]. Thus, the training should potentiate propulsive forces and reduce the opposing forces to the swimmers’ locomotion [10,12,13]. Active drag is one of the main swimming determinants and has been assessed to better understand the changes in the swimmer’s technical ability [14,15,16]. Research about master swimmers’ technique has focused mainly on the upper limbs’ simple kinematics, without evaluating the hydrodynamic characterization along the training period. In young swimmers, there is a lack of consensus in the data, as eight training weeks did not cause changes to the active drag or its coefficient [14], but one training week especially focused on technique improvement was enough to reduce the coefficient of active drag [17]. In highly trained swimmers, propelling efficiency and active drag remained constant during the Olympic Games preparatory season [18]. Despite the data scarcity, these outcomes suggest that training experience, performance level and age could differently influence hydrodynamic changes along a training period.

Considering the increasing engagement and competitiveness of master swimming, it is essential to further understand the main changes caused by training, not only in performance but also in technique, resulting in reduced hydrodynamic drag and increased efficiency. Measuring active drag has been a challenge because assessing the water-resistance on the swimmers’ propulsion when they are swimming is much more difficult than measuring it when they are gliding (i.e., passive drag [19]). The measuring active drag system (MAD-System) has been used throughout the years as a reliable methodology to assess this hydrodynamic variable in front crawl [18,20], but it is not usually applied to master swimming. The present study aimed to analyze the effects of a training mesocycle in master swimmers’ performance and hydrodynamic characteristics. It was hypothesized that a training period of four weeks, especially focused on aerobic training and centered on anaerobic conditioning and technique development, would result in sprint performance improvements and reduced active drag.

## 2. Materials and Methods

### 2.1. Participants

Twenty-two master swimmers (39.87 ± 6.10 years old, 1.74 ± 0.08 m height, 73.55 ± 13.63 kg body mass, 24.00 ± 3.00 kg·m^−2^ body mass index and 6.47 ± 5.41 years of experience) volunteered to participate in the study. Male (n = 16) and female (n = 6) swimmers were recruited by detailed announcements at a local swimming club using the following inclusion criteria: Male or female, aged 30–50 years old, engaged in a systematic swimming training program (two–three times per week, with a training volume ≥ 1000 m per session) with a master swimmer background in national swimming events. The criteria excluded swimmers who had a musculoskeletal injury, pathology or physical impairment in the previous six months, and who were absent ≥ 2 consecutive training units during the follow-up period. All subjects received detailed information on the study procedures and signed written informed consent. The study was approved by the local Institutional Review Board (project d975, December 2015) and was in accordance with the Declaration of Helsinki.

### 2.2. Experimental Procedures

A longitudinal research study was conducted with repeated performance measurements and drag-related variables being implemented before and after a four-week duration mesocycle. During this period, swimming training comprised three sessions per week (4.5 ± 0.9 km per microcycle), with low- to high-intensity aerobic and anaerobic swimming series and technical drills. Throughout the mesocycle, 89.5 ± 3.2% vs. 10.5 ± 3.2% of the total volume was performed at intensities corresponding to aerobic vs. anaerobic paces (4.7 vs. 0.6, 3.6 vs. 0.4, 3.0 vs. 0.5 and 5.0 vs. 0.4 km at weeks 1–4, respectively). The distinction between aerobic and anaerobic loads was carried out considering the specialized literature [6,7,21]. During the four-week training period, 8.6 ± 0.8% of the training tasks focused on swimming technique development (0.5, 0.3, 0.3 and 0.5 km at weeks 1–4, respectively). The mesocycle training was entirely water-based and no dryland components were performed. The training process was led by the team coach and accompanied by the research team.

Data collection was carried out under the same testing conditions, i.e., in a 25 m indoor swimming pool (with 27.5 °C water temperature and 60% relative air humidity), at the same time of the day and with no other swimmer(s) in the same lane or nearby lanes to reduce drafting and pacing effects. The experimental procedures consisted of two testing sessions on different days (24–48 h in between): (i) firstly, after 5 min of seated rest, body mass and height (Seca Instruments, Ltd., Hamburg, Germany) were measured, and body mass index (BMI) was calculated (dividing body mass by height squared); after a standard warm-up [22], each swimmer randomly performed a 25 m maximal trial or the active drag evaluation; (ii) in the second experimental session, the swimmers performed the same warm-up and were evaluated in the other 25 m test. All experiments were recorded in the swimmers’ sagittal plane using a stationary video camera operating at 50 Hz (HDR CX160E, Sony Electronics Inc., San Diego, CA, USA) positioned on the opposite side of the swimming pool. All swimmers were familiarized and experienced with the experimental apparatus. The swimmers consumed a similar diet, free of caffeine and alcohol, during the testing periods, and refrained from performing any strenuous physical activity for at least 24 h prior to the tests.

### 2.3. Performance Assessment in Free Swimming

Each swimmer performed two maximal 25 m front crawl bouts, starting with a push-off and resting 30 min in between, with the best trial used for further analysis [12,23]. Subjects reduced their gliding during the start so that data would be collected between the 11–24 m markers. A 50 Hz sampling rate electromechanical speedometer [24,25], connected through a cable to a harness belt attached to the swimmers’ waist, was used to assess linear kinematic variables. The speedometer was placed on the swimming pool forehead wall (~0.2 m above the water surface) and the corresponding hip velocity-time signals were shaped with a low-pass Butterworth filter with a 10 Hz cut-off frequency. A starting device was programmed to produce the starting signal and the velocimetric data output was backward synchronized with video images from the instant of take-off. The collected kinematical analysis of the 13 m data was performed using a MATLAB routine (version 2019, MathWorks, Inc., Massachusetts, USA), including a correction for the cable angulation effect [24,25].

The following linear kinematic variables were determined for each participant: (i) mean swimming speed (computed directly from the acquired speed data); (ii) maximum speed (obtained from the maximal value of instantaneous speed); (iii) minimum speed (corresponding to the minimal value of instantaneous speed); (iv) speed decrease (the speed decline from the 11 to the 24 m); and (v) hip horizontal intra-cyclic velocity variation (*dv*) were calculated as previously stated [13,26]:

(1) $$dv=\frac{\sqrt{\frac{\sum_{i}{\left(v_{i}-\bar{v}\right)}^{2}\times f_{i}}{n}}}{\frac{\sum_{i}v_{i}\times f_{i}}{n}}\times 100$$

in which *dv* represents the hip horizontal intra-cyclic velocity variation, *v* is the mean swimming speed, *v_i_* is the instant swimming speed, *f_i_* is the acquisition frequency and *n* is the of number speed–time pairs.

### 2.4. Active Drag Assessment

Each swimmer performed the 25 m at a constant speed on the MAD-System by pushing off with their upper limbs at the 16 fixed pads (maintaining their lower limbs elevated and constrained with a pull buoy) [15,18,20]. The push-off pads were attached to a 23 m rod (mounted 0.8 m below the water surface and with a 1.35 m distance between pads). The rod was instrumented with a force transducer to measure the push-off forces for each pad and the force signal was acquired using an A/D converter (Biopac, BIOPAC Systems, Inc., Goleta, CA, USA) at a 1000 Hz sample rate (and filtered with a 10 Hz cut-off frequency low-pass digital filter) [10,27]. The first and last pads were neglected to eliminate the influence of the swimmers’ wall push-off and their deceleration at the end of the 25 m bout. The force signal values from the remaining pads were time-integrated (giving the average force) and the mean speed was computed from the time taken to perform the distance between the second and the last pad. To establish the relationship between active drag and swimming speed, each participant completed 10 × 25 m lengths, ranging from minimal to maximal speeds (1.0–2.0 m·s^−1^, with 2 min intervals), with no differences larger than 0.01 m·s^−1^ from the imposed speed being observed [10,20,28]. For each length, mean drag force and mean swimming speed were measured, and the 10 speed/drag data was least-square fitted to the function using MATLAB [10,15,18]:

*D* = *k* × *v^n^* (2)

where *D* is the total active drag, *v* is the swimming speed and *k* and *n* are power function parameters [18,28]. The established fitted functions were used to calculate the active drag at 70%, 80%, 90% and 100% of the best mean speed obtained from the 25 m performance evaluation (individual best performance from pre- or post-training evaluation). The power to overcome drag (*W_d_*) was calculated as displayed in Equation (3) [10,28]. For a constant speed, the mean propelling force was considered to be equal to the mean drag force [18,20]:

*W_d_* = *D* × *v* (3)

### 2.5. Statistical Analysis

Standard statistical procedures were selected for the calculation of the means, standard deviations and 95% confidence limits. The normality of all distributions was verified by the Shapiro–Wilk test and it was observed that all the analyzed variables were homogeneous and normally distributed. Student’s paired *t*-test was used to compare pre- and post-training data, followed by Cohen’s d effect size with Hedge’s g correction. Effect size values of 0.20, 0.60, 1.20 and 2.00 corresponded to small, moderate, large and very large magnitudes, respectively [29]. All these statistical procedures were performed using IBM SPSS Statistics for Windows^®^, version 27.0 (Armonk, NY, USA: IBM Corporation), and the level of statistical significance was set at *p* ≤ 0.05.

## 3. Results

**Figure 1:**
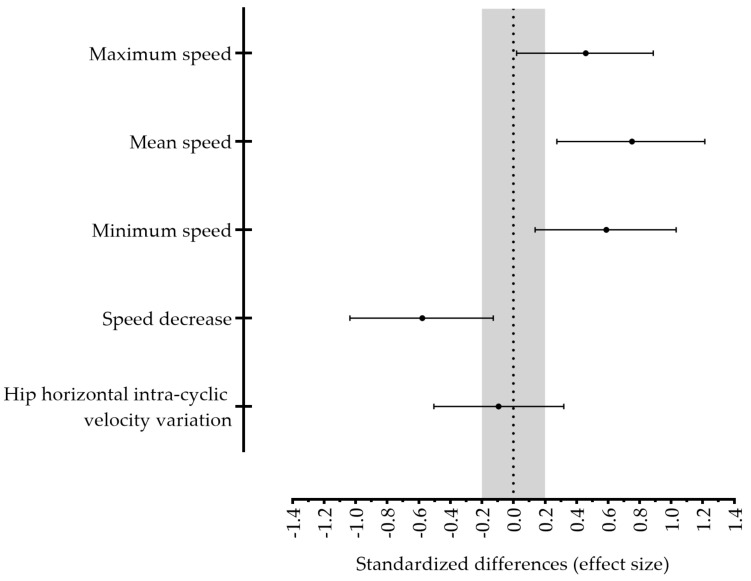
Performance-related variables standardized differences between post- and pre-training. The error bars indicate uncertainty in true mean changes with 95% confidence intervals.

Table 1 depicts the values of the performance-related variables measured before and after the training mesocycle. Higher maximum mean and minimum front crawl speeds were observed after the training period concurrently with a lower value of speed decrease. Hip horizontal intra-cyclic velocity variation did not evidence any pre- or post-training change. In addition, moderate to large beneficial training effects were found, except for intra-cyclic velocity variation (Figure 1).

The parameters obtained from the individually fitted curves during the evaluation of active drag are presented in Table 2.

**Figure 2:**
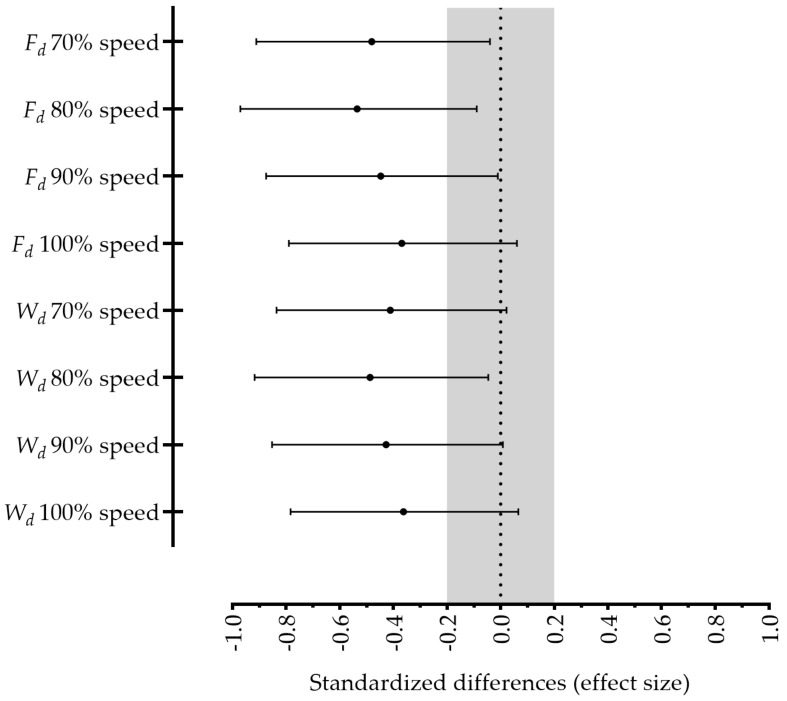
Standardized differences (effect size) between pre- and post-training regarding drag forces (*F_d_*) and power to overcome drag (*W_d_*) estimated from the 70%, 80%, 90% and 100% 25 m sprint speed.

The active drag and *W_d_* estimations at speeds corresponding to 70%, 80%, 90% and 100% of the sprint swimming speed are displayed in Table 3. A clear training effect on the active drag reduction at 70%, 80% and 90% of the 25 m maximum speed can be observed (mean ± 95% CI, −5.0 ± 3.9, 5.6 ± 4.0 and 5.9 ± 4.0%), but not at 100% exertion (5.9 ± 6.7%). Moreover, *W_d_* at 80% and 90% of the maximum speed decreased from pre- to post-training (5.6 ± 4.0 and 5.9 ± 5.2%, respectively). These data can also be observed by the standardized differences presented in Figure 2.

## 4. Discussion

This study aimed to assess the modifications induced by a four-week training mesocycle in master swimmers’ performance and hydrodynamic variables. Results showed that participants improved their front crawl sprint performance, particularly by increasing the 25 m mean, maximum and minimum speeds, as well as by lowering the speed decrease along the maximal bout. Nevertheless, the hip horizontal intra-cyclic velocity variation did not change with the training program. Regarding the swimmers’ main hydrodynamic characteristics, it was observed that the active drag and the power to overcome drag were improved at submaximal intensities (70%, 80% and 90% for drag, and 80% and 90% for *W_d_*) from pre- to post-training. These outcomes confirm the hypothesis that four weeks of aerobic swimming training, also focusing on anaerobic conditioning and technical development, would lead to positive performance changes and decrease master swimmers’ active drag.

Previous studies that focused on the training effect in master swimmers found improvements in energetic variables (e.g., oxygen uptake [30]), biomechanical indicators (e.g., stroke length and frequency, and propelling efficiency [4,6,7]), and race performance [30,31]. Past research also indicated that in this specific age group, performance throughout the season seems to be more dependent on technical factors [7], meaning that active drag reduction due to technical improvements would result in performance optimization. In fact, the observed performance enhancement identified in the present study was probably due to the active drag reduction, with swimmers reducing the power needed to propel through the water along the training mesocycle. In addition, the anaerobic training carried out in this study probably contributed to a better technique swimming performance at a higher speed. It is known that sprint performance tends to decrease with age in both men and women [32], but high-intensity and resistance training could be used to slow down speed decline or even to improve performance [33,34]. Possibly, these improvements could be greater if the swimmers had combined the in-water training with resistance training sessions, as suggested previously [33,34].

Previous findings in master swimmers suggested that eight training weeks improved stroke length, stroke index and stroke efficiency [7], which are efficiency indicators and can be used to evaluate swimming technical changes [5,10]. Moreover, it was identified that front crawl sprint performance (i.e., 15 m, 25 m, 50 m) is dependent on stroke index values in master swimmers of similar age (30–39 years old) to the ones in the current study [35]. Therefore, it seems to be a valid strategy for master swimmers to develop their technical skills concurrently with the conditional bioenergetic training [6,7], with these improvements being more evident at the beginning of the season [7]. Individual response to training depends to a great extent on the swimmers’ level, with higher gains when their experience and competitiveness are lower. The performance times of the current study’s participants are lower than elite-level swimmers and highly trained master swimmers [4,8], which means that their technical skills could suffer a larger enhancement, and consequently, their performance has a great margin for improvement. Additionally, the current sample included both male and female swimmers, and this could have influenced the results as it is known that men swim faster than women, although there is a tendency for this gap to be reduced after the age of 30 [36].

Hydrodynamic drag is a major swimming energy cost determinant [16,37] and is fundamental for optimizing performance. The current study’s outcomes evidenced that the four-week training mesocycle resulted in a significant reduction of the participants’ active drag, probably due to a reduction of lateral body movements and/or excessive kicking actions amplitude, as well as to a better-streamlined body position [16,38,39]. Technical training leads to a hydrodynamic resistance decrease in elite swimmers [40,41] by achieving a better-streamlined position and also by increasing the generation of lift and vortex forces to obtain a better progression through the water [42]. In this study, the main training effects were found at 80 and 90% of the individual sprint speed, both for the active drag and the power needed to overcome drag. This is explained by the fact that the major training volume was performed at submaximal intensities, although the anaerobic conditioning was also considered, with training adaptations enhancing body position and technique at those intensities [16]. Moreover, hydrodynamic improvements could also be a consequence of the swimming drills that occupied a significant part of the training program (~10%) [13].

Active drag depends on the fluid surface, pressure and wave effects on the swimmer’s body and on their center-of-mass velocity fluctuations [17,40,42]. Attempts to decrease the swimmers’ active drag have been made by shaving body hair and by using specific swimsuits, both aiming to reduce body surface resistance [43]. However, decreasing active drag by minimizing frontal area [37], particularly by achieving a better body alignment, reducing the lateral body movements (e.g., hand entry crossing and hips oscillation) and lowering the limbs range of motion is likely to have a larger effect on overall drag. This might be achieved by developing a better longitudinal body rotation and by using smoother movements while swimming–skills that might be improved by performing specific technical exercises and training series at competitive paces [44].

In the current study, some limitations should be addressed, such as the small sample size, the higher number of males than females, and the difference regarding the swimmers’ competitive experience. Some were former national-level swimmers and others started participating in competitive events more recently. This fact, along with the master swimmers’ work and familiar activities, implied a low weekly training frequency and volume compared to junior and senior swimmers, which might have negatively influenced their sprint performance. We also observed an eventual confounding factor of intra-subject variability, as evidenced in the active drag assessment, since the coefficient of proportionality for active drag measurement decreased from pre- to post-training in 13 swimmers while it was stable in the other 9. Thus, even knowing that it is novel to analyze master swimmers’ active drag and the training effect on their performance and hydrodynamics, the current results should be carefully analyzed. In fact, these data should be interpreted in the master swimming context and not transferred to other age groups and/or performance levels.

## 5. Conclusions

Master swimmers’ performance improved after a four-week training mesocycle centered on aerobic conditioning, also including anaerobic training series and technical development exercises. In ~35–45 year-old swimmers engaged in systematic training and regional and national-level competitive events, this training period was sufficient to cause relevant front-crawl hydrodynamic changes. These changes resulted in reduced drag forces during submaximal swimming, and consequently led to decreased power to overcome drag at these speeds. Centering the training process not only by swimming at low to moderate paces, but also potentiating anaerobic energy sources and enhancing the swimming technique might explain the obtained results. Future studies in this specific population should include: (i) other swimming determinants, particularly physiological (e.g., oxygen uptake, blood lactate and heart rate) and biomechanical variables (e.g., stroke frequency and length, and propelling efficiency) and (ii) other age groups (e.g., >50 years old) and training regimens. Other methods of active drag evaluation are also available, which might give complementary information to the active drag system used in this study.

## Figures and Tables

**Table 1:** Performance-related variables values assessed during the maximal 25 m front crawl pre- and post-training period of four weeks.

Variables	Pre-Training	Post-Training	% Change (Mean ± 95% CI)	*p*-Value
Maximum speed (m·s^−1^)	1.91 ± 0.36	1.96 ± 0.33	3.12 ± 2.76	0.04 *
Mean speed (m·s^−1^)	1.52 ± 0.25	1.57 ± 0.24	2.92 ± 1.60	0.01 **
Minimum speed (m·s^−1^)	1.17 ± 0.25	1.22 ± 0.25	4.64 ± 3.15	0.01 **
Speed decrease (%)	3.19 ± 3.42	1.05 ± 3.41	−82.46 ± 76.26	0.01 **
*dv* (%)	10.88 ± 4.09	10.73 ± 3.63	0.67 ± 6.30	0.66

Hip horizontal velocity intra-cyclic variation (*dv*). * and **: *p* ≤ 0.05 and 0.01.

**Table 2:** Values of the least-square fitted parameters describing the curves of the active drag dependent on swimming speed (*D* = *k × v^n^*).

Subject	Pre-Training			Post-Training		
	*k*	*n*		*k*	*n*	
A	64.50	1.44		58.13	1.77	
B	28.55	2.55		23.48	3.10	
C	36.45	2.41		38.04	2.37	
D	47.14	2.22		42.41	2.87	
E	29.68	2.35		28.40	1.81	
F	49.29	2.08		47.61	1.85	
G	35.50	2.99		37.20	2.93	
H	37.92	2.36		42.35	2.25	
I	43.54	2.32		43.78	1.42	
J	44.11	1.86		36.09	1.93	
K	39.07	2.28		34.05	2.90	
L	57.19	1.89		50.97	1.92	
M	38.25	2.63		39.62	1.94	
N	54.23	2.35		62.01	1.21	
O	62.27	1.50		60.29	1.56	
P	60.19	2.08		55.09	2.00	
Q	30.41	2.26		38.96	1.75	
R	26.43	2.22		23.67	2.36	
S	43.36	2.04		30.50	2.24	
T	49.86	1.89		48.01	2.53	
U	37.38	1.75		39.85	2.04	
V	30.69	1.99		31.47	1.56	

*k* = coefficient of proportionality, *n* = power of the speed.

**Table 3:** Drag and power variables obtained from the measuring active drag system at the pre- and post-training moments (according to the best free-swimming speed percentage).

Swimming Speed (%)		Active Drag (N)		*p*-Value	*W_d_* (W)		*p*-Value
		Pre-Training	Post-Training		Pre-Training	Post-Training	
70%	1.10 ± 1.17 m·s^−1^	53.68 ± 16.65	51.12 ± 17.16	0.03 *	60.80 ± 25.71	58.20 ± 26.62	0.06
80%	1.26 ± 0.19 m·s^−1^	71.78 ± 22.86	67.70 ± 22.49	0.02 *	93.22 ± 40.69	88.22 ± 40.53	0.03 *
90%	1.41 ± 0.21 m·s^−1^	92.92 ± 30.75	87.06 ± 29.71	0.05 *	136.13 ± 61.41	127.80 ± 59.98	0.05 *
100%	1.57 ± 0.24 m·s^−1^	117.22 ± 40.52	109.37 ± 39.24	0.09	191.25 ± 89.15	178.58 ± 86.50	0.10

Power to overcome drag (W*_d_*). * *p* ≤ 0.05.

## Data Availability

The data presented in this study are available on request from the corresponding author.
